# The growing need to establish a global wastewater surveillance consortium for future pandemic preparedness

**DOI:** 10.1093/jtm/taad035

**Published:** 2023-03-16

**Authors:** Michio Murakami, Masaaki Kitajima, Noriko Endo, Warish Ahmed, Bernd Manfred Gawlik

**Affiliations:** Center for Infectious Disease Education and Research, Osaka University, Suita, Osaka 565-0871, Japan; Division of Environmental Engineering, Faculty of Engineering, Hokkaido University, Sapporo, Hokkaido 060-8628, Japan; Research Center for Environmental Quality Management, Kyoto University, Otsu, Shiga 520-0811, Japan; CSIRO Environment, Ecosciences Precinct, 41 Boggo Road, Dutton Park, QLD 4102, Australia; European Commission, Joint Research Centre, Ispra 21027, Italy

**Keywords:** COVID-19, SARS-CoV-2, wastewater surveillance, pandemic preparedness, global monitoring, aircraft wastewater

## Abstract

Recognizing the risk of pandemic and the importance of monitoring and data sharing, we highlight the importance of establishing a global wastewater surveillance consortium, particularly under the umbrella of an international organization such as WHO, to strengthen future pandemic preparedness.

The deregulation of COVID-19 measures in Asian countries may lead to infection spread and a lack of fully notifiable clinical surveillance, thus making it difficult to ascertain the actual state of infection and identify the emergence and spread of novel variants. Wastewater surveillance is the most cost-effective means of assessing COVID-19 infection^1^ because infected individuals expel bodily waste and sheds SARS-CoV-2 into sewer systems. In Japan, where fully notifiable clinical surveillance has been conducted, a recent study using a highly sensitive viral RNA detection method demonstrated that SARS-CoV-2 could be detected from 50% of wastewater samples when daily newly reported COVID-19 cases were 0.69/100 000 inhabitants in the corresponding region and that the SARS-CoV-2 RNA concentration in wastewater showed a strong correlation with the number of reported cases (*r* = 0.94).^2^ Furthermore, viral genome sequencing can be used to identify specific variants.^3^ Wastewater monitoring for aircraft was proposed as a surveillance tool for assessing the presence of infected travelers^4^ and is underway for aircraft arriving from China/other countries in the USA, Australia and EU to understand the infection status and importation of emerging variants.^5^ Wastewater surveillance is applicable to a variety of pathogens including poliovirus, mpox (formerly known as monkeypox) virus and influenza virus as well as SARS-CoV-2.^1^

Given the need for a global surveillance network that provides early warnings against potential health threats, the role of wastewater surveillance is increasing rapidly as an approach for assessing the international transmission of SARS-CoV-2, its variants and other pathogens. To date, the monitoring system has evolved with the global outbreak of infectious diseases. The International Health Regulations were revised according to the experience from the SARS outbreak in 2002–03. In the revised regulations, it is mandated to share the status of infectious diseases under the umbrella of the WHO when a pathogen that may pose a public health emergency of international concern is identified. In this context, we propose here that wastewater surveillance should be incorporated into such international and regulatory framework as a global infectious disease surveillance system. Urban wastewater data generated in a geographical region can be used to prepare appropriate infection control measures in another geographical region. Furthermore, aircraft wastewater genomic surveillance can potentially track the transboundary pathways of emerging viruses. The EU, in collaboration with Canada, the USA, the UK, Malaysia, Australia and other countries, is establishing an international wastewater monitoring consortium by open data sharing regarding SARS-CoV-2 in urban and aircraft wastewater (https://wastewater-observatory.jrc.ec.europa.eu/). However, establishing an effective global wastewater surveillance consortium calls for the participation of more countries from a wide range of geographical areas and economic levels under G7 initiatives, and possibly under the umbrella of an international organization such as the WHO. Such a consortium could share experiences and protocols and support monitoring in low-income countries, for building a valuable public health tool for future pandemic preparedness.

## Data Availability

All relevant data are within the manuscript.
